# Marine Bacteriocins: An Evolutionary Gold Mine to Payoff Antibiotic Resistance

**DOI:** 10.3390/md22090388

**Published:** 2024-08-28

**Authors:** Piyush Baindara, Roy Dinata, Santi M. Mandal

**Affiliations:** 1 Animal Sciences Research Center, Division of Animal Sciences, University of Missouri, Columbia, MO 65211, USA; 2 Department of Biological Sciences, Indian Institute of Science Education and Research, Kolkata 741246, India; dinataroy9@gmail.com; 3 Department of Chemistry and Biochemistry, University of California San Diego, 9500 Gilman Dr, La Jolla, CA 92093, USA; mandalsm@gmail.com

**Keywords:** marine organisms, bacteriocins, lanthipeptides, RiPPs, evolution, drug resistance, marine ecosystem

## Abstract

The rapid evolution of drug resistance is one of the greatest health issues of the 21st century. There is an alarming situation to find new therapeutic strategies or candidate drugs to tackle ongoing multi-drug resistance development. The marine environment is one of the prime natural ecosystems on Earth, the majority of which is still unexplored, especially when it comes to the microbes. A wide variety of bioactive compounds have been obtained from a varied range of marine organisms; however, marine bacteria-produced bacteriocins are still undermined. Owing to the distinct environmental stresses that marine bacterial communities encounter, their bioactive compounds frequently undergo distinct adaptations that confer on them a variety of shapes and functions, setting them apart from their terrestrial counterparts. Bacterially produced ribosomally synthesized and posttranslationally modified peptides (RiPPs), known as bacteriocins, are one of the special interests to be considered as an alternative to conventional antibiotics because of their variety in structure and diverse potential biological activities. Additionally, the gut microbiome of marine creatures are a largely unexplored source of new bacteriocins with promising activities. There is a huge possibility of novel bacteriocins from marine bacterial communities that might come out as efficient candidates to fight against antibiotic resistance, especially in light of the growing pressure from antibiotic-resistant diseases and industrial desire for innovative treatments. The present review summarizes known and fully characterized marine bacteriocins, their evolutionary aspects, challenges, and the huge possibilities of unexplored novel bacteriocins from marine bacterial communities present in diverse marine ecosystems.

## 1. Introduction

The rapid emergence of drug resistance in pathogenic bacteria poses a catastrophic threat all around the world. At the same time, the unavailability of new drugs or anti-infective therapies creates an alarming situation concerning global health in the battle against drug resistance [1]. Overall, there is an urgent need for new antimicrobial or alternative strategies to combat drug-resistant pathogens. Microbial secondary metabolites consist of structurally diverse natural products which are essential phenomena of microbial communities, playing a diverse role in biotic and abiotic interactions [2]. Bacteriocins are one such secondary metabolite produced by bacterial communities in competitive complex environments such as soil, human gut, or marine sediments [3,4,5]. Bacteriocins are ribosomally synthesized and posttranslationally modified peptides (RiPPs) that have already been suggested as a viable alternative to conventional antibiotics [6,7]. As bacterial communities are highly diverse considering their intra- or inter-species interactions, bacteriocins are a rapidly evolving group of antimicrobial peptides (AMPs); however, a universal classification to accommodate all is yet to come. There are some proposed classifications available; however, many of the bacteriocins do not fit into them due to their novel and diverse structural properties [8,9]. Commonly, there are two types of bacteriocins, one containing an N-terminal leader sequence, and another one is leaderless bacteriocins. Leaderless bacteriocins are secreted in matured form, while the leader sequence containing bacteriocins are posttranslationally modified with conserved enzymes such as lanthionine synthetases where the leader sequence is cleaved off during the maturation process for the secretion of matured bacteriocins. Lanthionine-containing bacteriocins are designated as lanthipeptides, characterized by the presence of intramolecular thioether bridges, and turn out into complex polycyclic structures. Lanthipeptides are usually synthesized as a precursor peptide consisting of an N-terminal leader peptide and a C-terminal core peptide that serves as a substrate for the specific lanthionine synthetases. The posttranslational modification of lanthipeptides involves the dehydration of the selected serine and threonine residues to dehydroalanines (Dha) and dehydrobutyrines (Dhb) and a subsequent, intramolecular cyclization of nearby Cys thiols to the dehydrated residues (Dha or Dhb), forming lanthionine and methyl-lanthionine bridges, respectively, through a Michael-type addition. Finally, the posttranslationally modified precursor peptide moves to the cell membrane where the N-terminal leader sequence is cleaved off by the C39 protease domain of the ABC transporter and the matured modified core peptide is released to the extracellular space [10]. Interestingly, oligotrophic oceans are the largest ecosystem on Earth estimated to have 3.28 × 10^4^ to 2.46 × 10^6^ bacteria as amplicon sequence variants; however, little has been explored yet [11]. Enormous and unexplored bacterial diversity associated with diverse marine ecosystems suggested a huge potential for the availability of novel bacteriocins to fight against rapidly evolving drug-resistant bacteria. Therefore, in the present review, we have summarized the structural and physiochemical diversity of known and fully characterized marine bacteriocins along with their antimicrobial spectrum. Additionally, we have also discussed the possible evolutionary aspect of the relationship between lanthipeptides (lanthionine) and leaderless bacteriocins (non-lanthionine). Our sequence-based analysis suggested that marine bacteriocins are driven under a possible evolutionary pressure contributed by extreme marine conditions. Further, experimental pieces of evidence are required; however, the present analysis and review of available marine bacteriocins at least suggested a huge possibility of having new bacteriocins to payoff antibiotic resistance.

## 2. Diverse Bacteriocins from Marine Bacteria

### 2.1. Leaderless Bacteriocins

#### 2.1.1. Piscicocin

**Figure 1:**
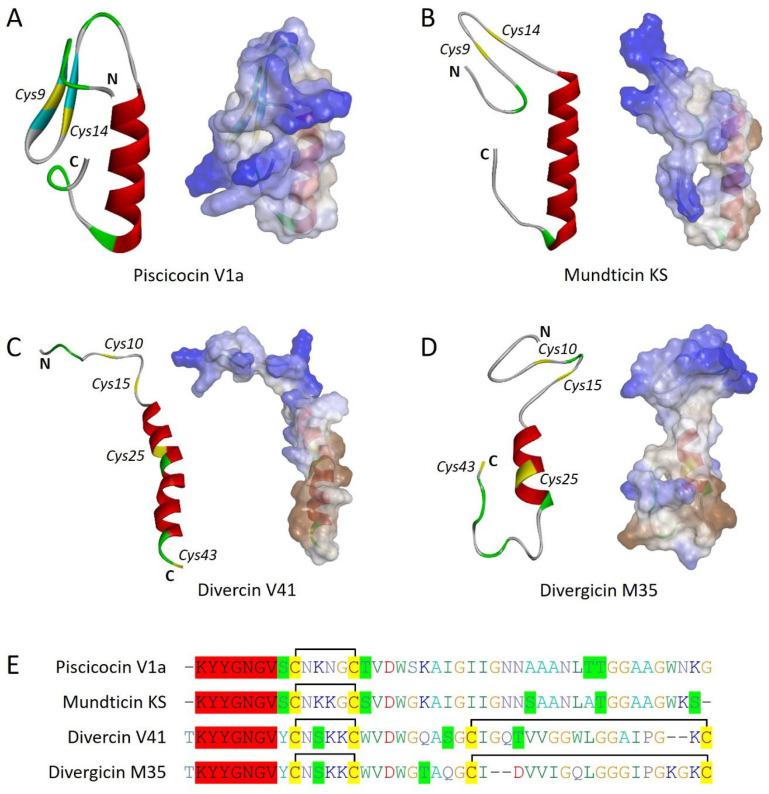
Marine bacteriocins (non-lanthionine) belonging to class IIa. (**A**) Piscicocin V1a. (**B**) Mundticin KS. (**C**) Divercin V41. (**D**) Divergicin M35. The left side of each panel shows solid ribbon structures and the left panel shows the overall surface charge of respective bacteriocins. Cysteine residues are numbered (italics) and highlighted in yellow color. N and C represent the N-terminal and C-terminal, respectively. Structures are predicted by using the SWISS-MODEL server. (**E**) Multiple sequences alignment (CLUSTALW) of class IIa marine bacteriocins. The conserved sequence at the N-terminal is highlighted in red color. The cysteine residues are highlighted in yellow color while the connecting black lines indicate the disulfide bonds. Nearby serine and threonine residues are highlighted in green color.

Piscicocins V1a (44 aa) and V1b (43 aa) are leaderless (non-lanthionine) bacteriocins belonging to class IIa. Piscicocins are produced by a lactic acid bacteria (LAB) *Carnobacterium piscicola* strain V1 which was originally isolated from fish [12]. Both V1a and V1b are reported to be produced in the cell-free supernatant of the same bacteria; however, their genetic organization is not known. V1a is reported as a novel bacteriocin while V1b is found similar to carnobacteriocin BM1, produced by *C. piscicola* LV17B; however, both contain the conserved motif YGNGV. Interestingly, V1a contained two cysteine residues at positions 9 and 14 that were confirmed to form a disulfide bond (Figure 1A,E) [13]. Piscicocins V1a displayed 100 times more potent activity than piscicocin V1b against the Gram-positive bacteria, though the activity spectrum was the same for both.

Piscicocin CS526 is another class IIa bacteriocin produced by *C. piscicola CS526* isolated from surimi (a fish-based product). It showed high similarity to piscicocin while the N-terminal conserved sequence is YGNGL rather than YGNGV where valine is replaced by leucine. It displayed activity against Gram-positive bacteria including *Enterococcus*, *Listeria*, *Pediococcus*, and *Leuconostoc* (Table 1) [14]. Different variants of piscicocins produced by different bacterial strains with a single amino acid mutation suggested an evolutionary force behind the production of distinct bacteriocins that suggests the possibility of having new bacteriocins as well. It will be interesting to explore and compare the genetic organization of piscicocins to further understand the evolutionary process. 

#### 2.1.2. Mundticin KS

Mundticin KS is a class IIa bacteriocin that was first reported to be produced by *Enterococcus mundtii* NFRI 7393, isolated from grass silage [15]. Later, Schelegueda et al. isolated and purified the same bacteriocins from *E. mundtii* Tw56, isolated from the intestine of a cold water marine fish, silverside (*Odontesthes platensis*) [16]. Mundticin KS is a leaderless, non-lanthionine bacteriocin with the motif YGNGV at the N-terminal, similar to other class IIa bacteriocins. Additionally, mundticin KS contains one disulfide bond between Cys9 and Cys14, which is well conserved among other class IIa bacteriocins (Figure 1B,E). Mundticin KS was found active against Gram-positive bacteria including different strains of *E. faecium*, *Lactobacillus plantarum*, *L. lactis*, *L. curvatus*, and *Listeria monocytogenes*; however, Schelegueda et al. reported later that the cell-free supernatant of *E. mundtii* Tw56 was also active against *Pediococcus pentosaceus*, *Streptococcus thermophiles*, *S. pyogenes*, and some Gram-negative bacteria, including *Pseudomonas aeruginosa* and *Shewanella putrefaciens* (Table 1) [15,16].

#### 2.1.3. Divercin V41

Divercin V41 is produced by *C. divergens* V41, isolated from fish viscera [12]. Divercin V41 is a non-lanthionine bacteriocin, belonging to class IIa; however, it is reported to have a 23 amino acid-long N-terminal sequence which has to be cleaved off to generate a 43 amino acid-long mature bacteriocin. Interestingly, the divercin V41 gene cluster was revealed to have two components of a lantibiotic-type signal transduction system at the same time and also have multiple cysteine residues along with adjacent serine or threonine residues (Figure 1C,E). This all provides a template for the synthesis of lanthionine bridges; however, lanthionine dehydrates and cyclases are absent in the gene cluster of divercin V41 [17]. This suggests an evolutionary position of divercin V41 between lanthipeptides and leaderless bacteriocins. Further, divercin V41 was confirmed to have two disulfide bonds between Cys10-Cys15 and Cys25-Cys43. Interestingly, divercin V41 demonstrates specific activity against Gram-positive bacteria (Table 1).

#### 2.1.4. Divergicin M35

Divergicin M35 is a class IIa bacteriocin (leaderless and non-lanthionine) produced by *C. divergens* M35 which is isolated from frozen smoked mussels. Divergicin M35 was revealed to have a molecular weight of 4518.75 Da (43 amino acids) consisting of 4 cysteine residues that are involved in disulfide bond formation (Cys10-Cys15 and Cys25-Cys43) while the conserved sequence YGNGV is present at the N-terminal like the other bacteriocins of the same class (Figure 1D,E). It is reported to have specifically strong activity against *L. monocytogenes* and could be potentially useful in food preservation (Table 1) [18].

#### 2.1.5. BaCf3

BaCf3 is a 27 amino acid-long, leaderless, non-lanthionine marine bacteriocin produced by *Bacillus amyloliquefaciens* BTSS3 isolated from the gut of deep-sea shark (*Centroscyllium fabricii*). BaCf3 was confirmed to have a molecular weight of 3028.42 Da with the presence of three cysteine residues while Cys6 and Cys13 formed a disulfide bond. Interestingly, the structure prediction of BaCf3 showed a high resemblance with laterosporulin that are highly similar to human beta-defensins [30]. Furthermore, BaCf3 also showed anticancer activities similar to laterosporulin10 [31]. BaCf3 was reported to inhibit the biofilm formation by different Gram-positive bacteria including different *Bacillus* sp., *S. warnie*, *Micrococcus luteus*, *Geobacillus stearothermophilus*. Additionally, it displayed potential activity against food pathogens including *Salmonella typhimurium*, *Clostridium perfringens*, and *E. faecalis* (Table 1) [19]. Interestingly, BaCf3 did not show any similarities with any existing class of bacteriocins that suggests the presence and possibilities of new bacteriocins in marine ecosystems. 

#### 2.1.6. Sonorensin

**Figure 2:**
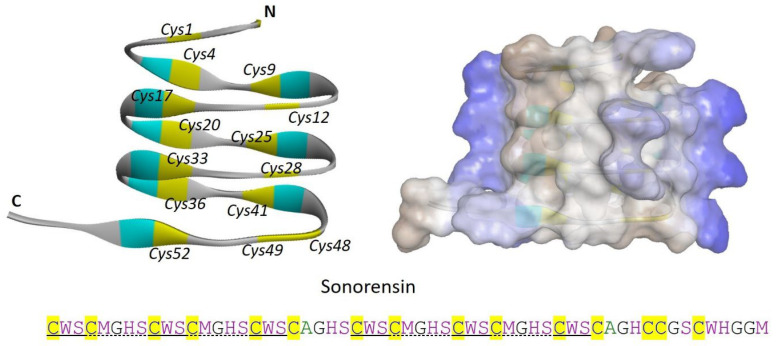
Sonorensin is an unusual marine bacteriocin with no class designation. The left side shows a solid ribbon structure and the left panel shows the overall surface charge of sonorensin. Cysteine residues are numbered (italics) and highlighted in yellow color. N and C represent the N-terminal and C-terminal of the sonorensin. The structure is predicted by using the SWISS-MODEL server. The cysteine residues are highlighted with yellow color in the amino acid sequence while the solid and dotted black lines under the amino acid sequence show the repeated motifs.

Sonorensin is a 57 amino acid-long, non-lanthionine, cysteine-rich bacteriocin produced by a marine bacterial isolate *B. sonorensis* MT93. The characterization of sonorensin does not provide sufficient proof to categorize it into any designated class of bacteriocins, while it have 53 amino acid-long leader sequence which is cleaved off during the production of the mature peptide. Unusually, sonorensin contains 15 cysteine residues, however, not characterized to be involved in any disulfide bond formation (Figure 2). Interestingly, sonorensin showed a broad activity spectrum against both Gram-positive and Gram-negative bacteria including *L. monocytogenes*, *V. vulnificus*, *B. subtilis*, *S. aureus*, *P. aeruginosa*, and *E. coli* (Table 1) [20]. Interestingly, sonorensin suggests a distinct class of bacteriocins with the presence of an unusually high number of cysteine residues. It seems interesting to explore the marine bacterial genomes to find out the sonorensin-like bacteriocins or new classes of related bacteriocins with potential bioactivities. 

#### 2.1.7. CAMT6

CAMT6 is a small (12 amino acids), leaderless, non-lanthionine bacteriocin produced by *E. durans* strain YQ-6, isolated from a marine fish, *Larimichthys polyactis*. Interestingly, CAMT6 is a unique bacteriocin itself that does not show similarity with other bacteriocins and thus represents a new class of bacteriocins. Additionally, it shows a poor similarity with surfactant-associated anionic peptides from sheep. CAMT6 shows the potential antimicrobial activities against both Gram-positive and Gram-negative bacteria including *S. aureus*, *B. subtilis*, *B. equi*, *B. cereus*, *S. haemolyticus*, *Propionibacterium acnes*, *S. paratyphi*, *V. parahaemolyticus*, *P. foulis*, and *Enterobacter aerogenes*. Interestingly, CAMT6 was also reported to disrupt biofilm formation by *L. monocytogenes* showing its potential applications in the food industry (Table 1) [21]. It is worth mentioning here that CAMT6 is extremely short and the only known marine bacteriocin with no cysteine residues. 

### 2.2. Lanthipeptides

#### 2.2.1. Nisin Z

**Figure 3:**
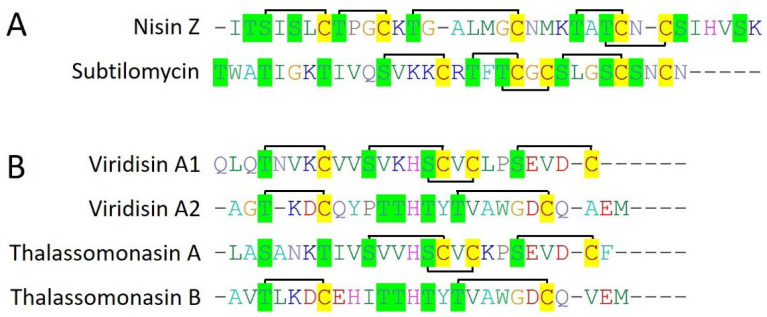
Known and characterized marine bacteriocins of class I (Type I lanthipeptides). Multiple sequence alignment (CLUSTALW) of type I lanthipeptides produced by Gram-positive bacteria (**A**), and (**B**) by Gram-negative bacteria. The cysteine residues are highlighted in yellow color while the serine and threonine residues are highlighted in green color. The connecting black lines indicate the ring topologies of lanthionine bonds.

Nisin Z (34 amino acids) belongs to class I bacteriocins (Type I lantibiotic), and was originally isolated from *Lactococcus lactic* NIZO 22186. Nisin Z turned out as a natural variant of nisin A, with a single mutation from histidine to asparagine at position 27 [32]. In the recent past, nisin Z production has also been reported by the bacterial isolates from the gut of marine fish [22,23]. Nisin Z contains 23 amino acid-long leader peptides which are cleaved off by peptidase (NisP) while mature nisin Z is released after the formation of lanthionine and methyllanthionine bridges by the respective class-specific lanthionine dehydratase (LanB) and lanthionine cyclases (Figure 3A) (LanC) [32]. It is reported that nisin Z produced by a bacterial isolate (*L. lactis* sub sp. lactis) from marine fish (olive flounder) showed efficient antimicrobial activity against *S. iniae* when prepared in 3.5% (*w*/*v*) NaCl (equivalent to seawater) [23]. Another study demonstrated that nisin Z produced by *L. lactis* TW34 (isolated from marine fish) effectively kills the fish pathogen *L. garvieae* (Table 1) [22]. Overall, it suggested that nisin Z is more adapted to its original habitat concerning the displayed antimicrobial activity and thus could be a potential candidate for seafood preservation or aquaculture pathogens.

#### 2.2.2. Subtilomycin

Subtilomycin is a 32 amino acid-long, class I bacteriocin (Type I lantibiotic) produced by a marine bacteria *B. subtilis* MMA7, isolated from the marine sponge *Haliclona simulans*. It possesses a 24 amino acid-long N-terminal leader peptide which is cleaved off during the peptide maturation by a peptidase present within the gene cluster. Subtilomycin contained five cysteine residues which all are involved in the formation of five lanthionine or mehthylanthionine rings subsequently after dehydration and cyclization by LanB and LanC, respectively (Figure 3A). Subtilomycin displayed potent activity against both Gram-positive and Gram-negative bacteria including different species of *Bacillus* and *Clostridium*, *L. monocytogenes*, *S. aureus*, and *P. aeruginosa*. Interestingly, subtilomycin has also been reported to inhibit the growth of vancomycin-intermediate *S. aureus* (VISA), methicillin-resistant *S. aureus* (MRSA), vancomycin-resistant *E. coli*, and different pathogenic *Candida* species (Table 1) [24].

#### 2.2.3. Viridisin

Viridisin is a class I bacteriocin (Type I lantibiotic) produced by marine bacteria *Thalassomonas viridans* XOM25. Viridisin is an unusual lanthipeptide as it consists of three core lanthipeptides in the gene cluster, VdsA1, VdsA2, and VdsA3 which have four, two, and two cysteine residues, respectively (Figure 3B). While corresponding lanthipeptides for VdsA1 (25 amino acids) and VdsA2 (25 amino acids) are cloned, expressed, and fully characterized for their lanthionine and methlyllanthione ring patterns, their antimicrobial activities are still needed to be explored [25]. The presence of three different core peptides in a single gene cluster suggests the preparedness of bacteriocins to compete with other close or distant organisms in adverse conditions. It will be interesting to further investigate the antimicrobial properties of different viridisins concerning structural variations.

#### 2.2.4. Thalassomonasin

Thalassomonasin is produced by a marine proteobacterium *T. actiniarum* NBRC 104231 and belongs to class I bacteriocins (Type I lantibiotic). Thalassomonasin is a two-component lanthipeptide consisting of two core lanthipeptide precursor genes, *tln* A1 (thalassomonasin A) and *tln* A2 (thalassomonasin B) in its gene cluster. Thalassomonasin A (25 amino acids) and Thalassomonasin B (26 amino acids) consist of three and two cysteine residues, respectively, which all are involved in the formation of lanthionine rings. Both thalassomonasin A and B have an N-terminal leader sequence of 29 amino acids that differ from each other, however, having characteristic conserved motifs for type I lanthipeptide leader sequences (Figure 3B). Interestingly, thalassomonasin A showed efficient activity against both Gram-positive and Gram-negative bacteria including *B. subtilis*, *S. aureus*, *M. luteus*, *E. coli*, and *P. aeruginosa* while Thalassomonasin B showed minor activity and thus was not explored further (Table 1) [26]. Thalassomonasin is another example of bacteriocin structural variation within the single gene cluster oriented for better survival in adverse conditions. 

#### 2.2.5. Formicin

**Figure 4:**
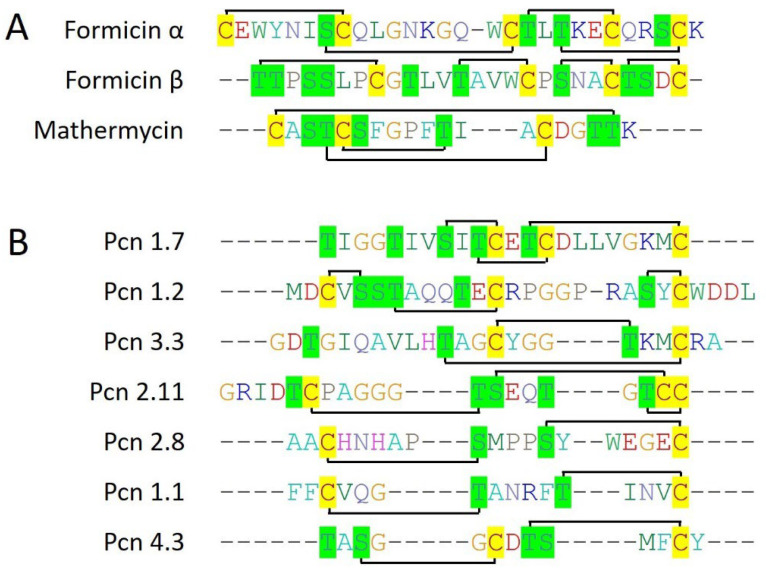
Known and characterized marine bacteriocins of class I (Type II lanthipeptides). Multiple sequence alignment (CLUSTALW) of type II lanthipeptides produced by Gram-positive bacteria (**A**), and (**B**) by Gram-negative cyanobacteria. The cysteine residues are highlighted in yellow color while the serine and threonine residues are highlighted in green color. The connecting black lines indicate the ring topologies of lanthionine bonds.

Formicin is a two-component bacteriocin (Type II lantibiotic) that belongs to class I bacteriocins. Formicin is produced by an antimicrobial-producing bacteria, *B. paralicheniformis* strain APC 1576, isolated from the intestine of Atlantic mackerel (*Scomber scombrus*), a marine fish. Formicin contains a 40 amino acid N-terminal leader sequence which is cleaved by LanP, residing within the gene cluster itself. As formicin is a type II lanthipeptide, its gene cluster consists of a bifunctional enzyme that performs both dehydration and cyclization subsequently during the maturation process of lanthipeptide before the cleavage of the leader peptide. Additionally, an overall +2 positive charge along with less hydrophobicity make formicin unique among all the type II lanthipeptides (Figure 4A). Interestingly, two core peptides within the single gene cluster, formicin α and formicin β, consisted of five and four cysteine residues, respectively, and were structurally different from each other, suggesting an evolutionary force shaping the bacteriocins according to the surrounding environment. Formicin showed potential antimicrobial activity against Gram-positive pathogenic strains including *L. monocytogenes*, *S. aureus*, *S. mutans*, *Clostridioides difficile*, *Clostridia*, and different species of *Enterococcus* (Table 1) [27].

#### 2.2.6. Mathermycin

Mathermycin is produced by marine actinomycete *Marinactinospora thermotolerans* SCSIO 00652 (isolated from sea marine sediments), a 19 amino acid-long class I bacteriocin (Type II lantibiotic). It contains a 60 amino acid-long N-terminal leader sequence and 3 cysteine residues that all are involved in the formation of lanthionine and methyllanthionine rings with nearby serine and threonine residues (Figure 4A). Mathermycin showed a closed structural homology with cinnamycin and duramycin. Also, mathermycin was revealed to have a similar activity spectrum against Gram-positive bacteria such as *B. subtilis*, like cinnamycin (Table 1) [28]. 

#### 2.2.7. Prochlorosins

Prochlorosins are a structurally diverse set of class I bacteriocins (Type II lantibiotic) produced by marine picocyanobacteria, *Prochlorococcus,* and *Synechococcus*. Surprisingly, picocyanobacteria employed an unusual mechanism that was able to produce structurally diverse lanthipeptides abundantly using a single lanthionine synthetase. Using a deep sequencing methodology, 50 *Prochlorococcus* and 26 *Synechococcus* genomes were analyzed for the presence of lanthionine synthetases and lantibiotic precursor peptides. Out of all, *Prochlorococcus MIT0701*, *Prochlorococcus MIT1327*, *Prochlorococcus MIT9303*, and *Prochlorococcus MIT9313* are found to have 9, 13, 13, and 29 while *Synechococcus WH8016*, *Synechococcus RS9916*, *Synechococcus KORDI100*, *Synechococcus MITS9508*, and *Synechococcus MITS9504* are found to have 1, 19, 9, 8, and 80 core lantibiotic sequences. In total, these 9 genomes were revealed to have 181 diverse core lanthipeptide genes which are almost double the total other lanthipeptides (~90) that have been reported from other different bacterial species [29]. The most structurally diverse lanthipeptides are observed in *Prochlorococcus MIT9313* and seven of them have been fully characterized for their ring topologies (Figure 4B). Overall studies suggested an unexplored treasure of bacteriocins in marine cyanobacteria going under a huge evolutionary pressure and could have been provided a large number of novel bacteriocins with potential therapeutic properties.

## 3. Evolutionary Diversity of Marine Bacteriocins

As the marine ecosystem is enormously diverse, a huge diversity of the marine bacterial communities and thus the marine bacteriocins is also anticipated. However, only a few bacteriocins belonging to defined classes have been reported from the marine ecosystem, representing a diverse group of AMPs (Figure 1, Figure 2, Figure 3 and Figure 4). Moreover, some of the bacteriocins did not fit into any defined class of bacteriocins which suggested the possibility of having novel classes of bacteriocins from the marine environment in the future. For example, BaCf3 is characterized as a leaderless bacteriocin with the unusual presence of three cysteine residues [19]. BaCf3 did not have any conserved sequence or show similarity with a known class of bacteriocins; however, structural resemblance showed some similarities with laterosporulins, a class IId bacteriocins produced by the different strains of *Brevibacillus laterosporus* [30,33]. Next, sonorensin contains 15 cysteine residues that make it unusually unique on its own and do not fit into any existing class of bacteriocins [20]. Additionally, CAMT6 is a small marine bacteriocin with no cysteine residues that do not resemble any existing class of bacteriocins [21]. These are just three bacteriocins with distinct structural features when compared to the existing classes of bacteriocins while the marine bacterial diversity is almost unexplored yet. The possibility of having multiple novel bacteriocins of new classes from the marine ecosystem can be easily estimated. 

**Figure 5:**
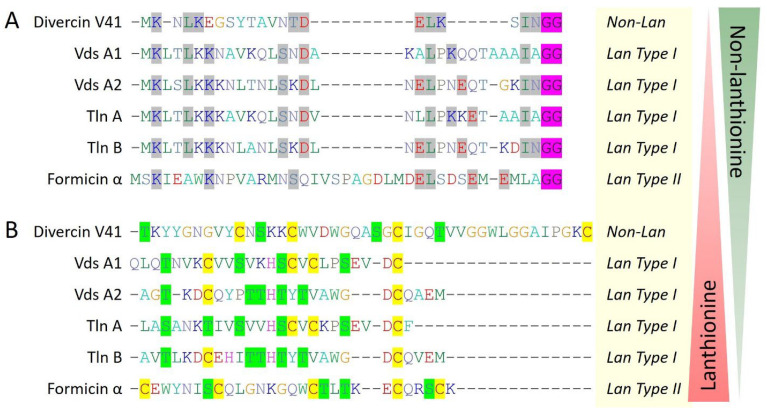
Multiple sequence alignment (CLUSTALW)-based hypothetical model for the evolution of marine bacteriocins from non-lanthionine to lanthionine containing lanthipeptides and vice versa. (**A**) Divercin V41 belongs to class IIa while unusually having an N-terminal leader sequence containing a double glycine motif (GG, highlighted in pink color), similar to lanthionine containing type I and type II lanthipeptides. Leader sequence alignment showing conserved residues (highlighted in gray color) suggesting divercin V41 is under evolutionary transition towards lanthipeptides or vice versa. (**B**) The core peptide sequence alignment showing diverse structural ring topologies. The cysteine residues are highlighted in yellow color while the serine and threonine residues are highlighted in green color.

Other four marine bacteriocins (piscicocin V1a, mundticin KS, divercin V41, and divergicin M35) belong to class IIa of bacteriocins and all have a disulfide bond followed by characteristic conserved sequence YGNGV at N-terminal. Notably, divercin V41 and divergicin M35 consist of two additional cysteine residues involved in disulfide formation (Figure 1). It seems there is an evolutionary pressure for the addition of an extra disulfide bond to make these bacteriocins more stable in adverse marine conditions for better survival of the producer. Additionally, the presence of two disulfide bonds further makes them structurally closer to eukaryotic defensins that have characteristic three disulfide bonds. Interestingly, divercin V41 was reported to have an N-terminal leader sequence that cleaved off during the maturation process. An N-terminal leader sequence, double glycine motif at the cleavage site, and presence of four cysteine residues along with serine and threonine residues suggest divercin V41 resemblance with lanthipeptides, where lanthionine synthetases are absent (Figure 5). 

Other than class IIa bacteriocins, six type I lanthipeptides (nisin Z, subtilomycin, viridisin A1, viridisin A2, thalassomonasin A, and thalassomonasin B) belonging to class I bacteriocins have been characterized from the marine bacteria. These bacteriocins contained 2 to 5 cysteine residues which all are involved in lanthionine ring formation with different topologies, making them structurally diverse (Figure 3). Next, formicin and mathermycin (type II lanthipeptides) were reported from the marine bacteria and consisted of 3 to 5 cysteine residues that involved different ring topologies (Figure 4). Additionally, 181 type II lanthipeptide genes were identified from gnome mining of the 9 cyanobacteria genomes isolated from the deep sea [29]. This provides a glimpse of the huge unexplored diversity of marine ecosystems and the possibility of having plenty of novel and diverse bacteriocins from the underexplored marine ecosystem. Out of all, *Prochlorococcus MIT9313* alone was reported to have 29 lanthipeptide gene clusters, out of which 7 most interesting and diverse lanthipeptides have been characterized from the genome (Figure 4). Surprisingly, ring topologies of these seven peptides were highly dissimilar to all the other known lanthipeptides and even with each other as all of the serine, threonine, or cysteine (2 to 3) residues followed unique positioning patterns while the leader sequences were highly conserved. This suggested an advanced evolutionary mechanism in marine cyanobacteria that can generate such a high extent of structural diversity of lanthipeptides. Overall, this indicates marine bacterial communities are residing under an ongoing evolutionary pressure that guides a unique mechanism of the structural diversification of marine bacteriocins. It can be hypothesized that with this huge diversity of different life domains and associated diverse bacterial communities under different physiological and physical conditions within marine ecosystems, what would be the extent of bacteriocin novelty and structural diversity which has been only a little bit explored yet?

## 4. Challenges and Future Directions

**Figure 6:**
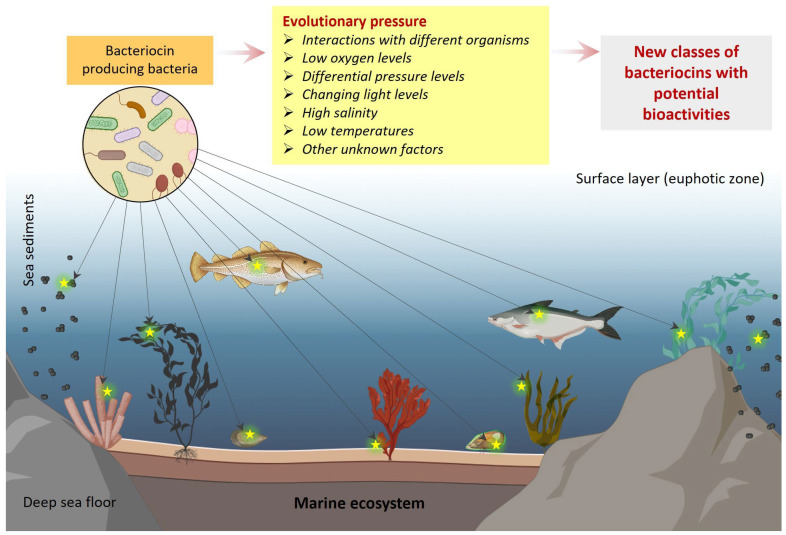
Graphical representation showing the diverse interactions of bacteriocin-producing bacteria with different host organisms and free marine sediments present within the marine ecosystem. The yellow glowing star represents the interacting source organism or the place of bacteriocin-producing bacteria.

Marine habitats are the largest and one of the most diverse ecosystems on the Earth which has only been explored a little bit yet and much is still far away from human interference. One of the biggest challenges is to access or analyze the vast marine diversity and thus the biodiscovery of its associated bacterial communities and bacteriocins. Also, marine bacterial communities represent a dilute habitat which is an unusual place for the action of bacteriocins. Next, whatever marine bacterial communities we have explored yet are incomplete as many of them are uncultivable in lab conditions unless created in very similar conditions to their native marine habitats. This is why the bacteriocins biosynthetic gene clusters of marine bacteria are not fully expressed in the in vitro lab conditions and remain inaccessible [34]. Additionally, marine habitats are physically diverse such as sea cost subsurface soil, sea cost sediments, and deep-sea sediments; moreover, the marine ecosystem is divided into five different zones based on different environment pressures, light levels, temperatures, and dissolved oxygen concentration, and thus results in diverse microbial communities [35]. Especially low oxygen levels in the deep-sea environment create anaerobic conditions that significantly affect the antimicrobial production by the deep-sea bacteria [36,37]. All these various factors play essential roles and affect the secondary metabolites and bacteriocins produced by marine bacterial communities (Figure 6) [38,39]. The design and development of more sophisticated culture conditions or strategies are required to recover the uncultivable marine bacterial communities and thus the diversity of diverse unexplored bacteriocins. Most of the reported marine bacteriocins are from bacterial species isolated either from the gut or in association with the other marine organisms that again suggested the little-known information about the marine bacteriocins. Additionally, each organism has its specific gut microbiome, where bacteriocin-producing bacteria interact with the other prokaryotic organisms within the gut and the eukaryotic host as well [40]. These diverse interactions between different microbial communities from different sea organisms under the diverse conditions of different marine habitats generate a huge evolutionary pressure for the resulting structural diversity of bacteriocins or other secondary metabolites that still need to be explored. Moreover, lanthipeptide (class I bacteriocins) modifying enzymes lanthionine synthetases are known to play a role as signaling molecules in bacterial communities that suggest their diverse functions in bacterial cross-talk and might be a factor regulating the production of specific bacteriocins [41]. 

Next, in the recent past, culture-independent techniques such as genome mining, metagenomics, next-generation sequencing, metagenomic library preparation, and metabolomics have been employed to explore inaccessible microbial communities including the discovery of new bacteriocins [42]. Thanks to the advancement of technology, however, sample collection itself is a big challenge in marine habitats such as the deep sea. Next, there are a few interesting genome mining tools available including BAGEL and antiSMASH, which can find out bacteriocins gene clusters based on the similarity basis of the available data sets that include conserved modifying enzymes and motif sequences within the leader or sometimes in core peptides [43,44]. Additionally, DeepRiPP and RODEO are other machine learning-based bioinformatics tools that can perform the high-throughput identification and classification of RiPPs [45,46]. Although genome mining tools have proven their potential in finding novel bacteriocins, the search is based on the similarities of the earlier identified RiPPs and might fail to detect novel posttranslational modification in the RiPPs, which are still need to be studied in detail while not available in data sets [47,48].

Even after, the identification and mining of bacteriocin-producing gene clusters using in silico genome mining, the gene cluster should be expressed in the native host for the subsequent production and purification of the respective bacteriocins, which is challenging. Moreover, it is reported that many of the bacteriocin gene clusters are “silently” present in the genomes, while the gene can be identified by metagenomic approaches but the biological activity is missing during experimental culture conditions [49,50]. Heterologous gene expression is one of the reliable and economical strategies for the controlled expression of these silent bacteriocins gene clusters [51]. Another strategy is the chemical synthesis of bacteriocins as peptidic in nature; however, chemical synthesis is expensive and not suitable to achieve the desired posttranslational modification such as lanthionine rings and disulfide bonds. As of the current scenario, a combination of various in silico and in vitro experiments with a defined strategy is the most acceptable approach for the identification and characterization of marine bacteriocins from free marine sediments as well as from the gut of marine organisms residing within the marine ecosystem.

## 5. Conclusions

In the current scenario of rapidly evolving drug resistance and the scarcity of new alternative drugs or strategies to fight against, there is a pressing need for novel bioactive molecules with diverse and unique mechanisms of action. Marine bacteriocins are one of the exciting and enormous groups for such bioactive molecules. Interestingly, due to its huge diversity, physical conditions, and several other factors, marine ecology is profoundly diverse, and so their bioactive compounds including bacteriocins; however, a little bit of it has been explored yet. Other than the free marine bacterial communities, marine organism’s gut microbiome is supposed to be more diverse due to interactions with marine hosts and then the overall marine ecology that deals with the high salinity, low temperatures, low oxygen or anaerobic conditions, and hydrostatic pressure. Overall, all of these factors create a cumulative evolutionary pressure for the marine bacterial communities that is reflected in the diverse structural and functional diversity of marine bacteriocins that largely remain unexplored (Figure 6). Limited accessibility for sample collection and technological barriers to analyzing the huge diversity are the major factors in the way of the true exploration of marine bacteriocins. In conclusion, marine bacteriocins could be a promising alternative to fight against drug-resistant pathogens; however, the true diversity and potential of marine bacteriocins are yet to be explored.

## Figures and Tables

**Table 1:** Details of known and fully characterized marine bacteriocins along with their respective class, producer bacterial strains, sources, and activity spectrum.

AMP	Class	Producer Bacteria	Source	Activity Spectrum	References
Leaderless					
Piscicocins V1a Piscicocin CS526	Class IIa	*C. piscicola* V1 *C. piscicola CS526*	Fresh fish, smoked and marinated fish, fish intestinal tract	*L. monocytogenes* *L. sake* *L. curvatus* *L. plantarum* *L. mesenteroıdes* *P. acidilactici* *E. faecalis*	[12,14]
Mundticin KS	Class IIa	*E. mundtii* NFRI 7393	Silverside (*Odontesthes platensis*)	*E. faecium* *L. plantarum* *L. lactis* *L. curvatus* *L. monocytogenes* *P. pentosaceus* *S. thermophiles* *S. pyogenes* *P. aeruginosa* *S. putrefaciens*	[15,16]
Diversin V41	Class IIa	*C. divergens* V41	Fish viscera	Gram-positive bacteria	[12,17]
Divergicin M35	Class IIa	*C. divergens* M35	Frozen smoked mussels	*L. monocytogenes*	[18]
BaCf3	Not assigned	*B. amyloliquefaciens* BTSS3	Deep-sea shark (*Centroscyllium fabricii*)	*Bacillus* sp. *S. warnie* *M. luteus* *G. stearothermophilus* *S. typhimurium* *C. perfringens* *E. faecalis*	[19]
Sonorensin	Not assigned	*B. sonorensis* MT93	Marine	*L. monocytogenes* *V. vulnificus* *B. subtilis* *S. aureus* *P. aeruginosa* *E. coli*	[20]
CAMT6	Not assigned	*E. durans* YQ-6	Marine fish (*Larimichthys polyactis*)	*S. aureus* *B. subtilis* *B. equi* *B. cereus* *S. haemolyticus* *P. acnes* *S.paratyphi* *V. parahaemolyticus* *P. foulis* *E. aerogenes* *L. monocytogenes*	[21]
Lanthipeptides					
Nisin Z	Type I lantibiotic	*L. lactic* NIZO 22186	Marine fish (Olive flounder)	*S. iniae* *L. garvieae*	[22,23]
Subtilomycin	Type I lantibiotic	*B. subtilis* MMA7	Marine sponge (*Haliclona simulans*)	*Bacillus* sp. *Clostridium* *L. monocytogenes* *S. aureus* *P. aeruginosa* VISA MRSA *Candida* sp.	[24]
Viridisin	Type I lantibiotic	*T. viridans* XOM25	Marine	Not determined	[25]
Thalassomonasin A and B	Type I lantibiotic	*T. actiniarum* NBRC 104231	Marine	*B. subtilis* *S. aureus* *M. luteus* *E. coli* *P. aeruginosa*	[26]
Formicin	Type II lantibiotic	*B. paralicheniformis* APC 1576	Atlantic mackerel (*Scomber scombrus*)	*L. monocytogenes* *S. aureus* *S. mutans* *C. difficile* *Clostridia* *Enterococcus* sp.	[27]
Mathermycin	Type II lantibiotic	*Marinactinospora thermotolerans* SCSIO 00652	Marine sediments	*B. subtilis*	[28]
Prochlorosins	Type II lantibiotic	*Prochlorococcus* *Synechococcus*	Marine	Not determined	[29]

VISA: vancomycin-intermediate *S. aureus*, MRSA: methicillin-resistant *S. aureus*.

## Data Availability

There is no additional data associated with this work.
